# Long-term effects of STN DBS on mood: psychosocial profiles remain stable in a 3-year follow-up

**DOI:** 10.1186/1471-2377-8-43

**Published:** 2008-11-11

**Authors:** Iris Kaiser, Ilse Kryspin-Exner, Thomas Brücke, Dieter Volc, François Alesch

**Affiliations:** 1 Department of Neurosurgery, Medical University of Vienna, Austria; 2 Department of Clinical and Health Psychology, Institute of Psychology, University of Vienna, Austria; 3 Department of Neurology, Wilhelminenspital, Vienna, Austria; 4 Department of Neurology, Confraternität, Vienna, Austria

## Abstract

**Background:**

Deep brain stimulation of the subthalamic nucleus significantly improves motor function in patients with severe Parkinson's disease. However, the effects on nonmotor aspects remain uncertain. The present study investigated the effects of subthalamic nucleus deep brain stimulation on mood and psychosocial functions in 33 patients with advanced Parkinson's disease in a three year follow-up.

**Methods:**

Self-rating questionnaires were administered to 33 patients prior to surgery as well as three, six, twelve and 36 months after surgery.

**Results:**

In the long run, motor function significantly improved after surgery. Mood and psychosocial functions transiently improved at one year but returned to baseline at 36 months after surgery. In addition, we performed cluster and discriminant function analyses and revealed four distinct psychosocial profiles, which remained relatively stable in the course of time. Two profiles featured impaired psychosocial functioning while the other two of them were characterized by greater psychosocial stability.

**Conclusion:**

Compared to baseline no worsening in mood and psychosocial functions was found three years after electrode implantation. Moreover, patients can be assigned to four distinct psychosocial profiles that are relatively stable in the time course. Since these subtypes already exist preoperatively the extent of psychosocial support can be anticipatory adjusted to the patients' needs in order to enhance coping strategies and compliance. This would allow early detection and even prevention of potential psychiatric adverse events after surgery. Given adequate psychosocial support, these findings imply that patients with mild psychiatric disturbances should not be excluded from surgery.

## Background

Parkinson's disease (PD) is a progressive movement disorder ensuing from dopaminergic depletion of the basal ganglia, substantia nigra pars compacta. The resulting disruption of the motor circuit that connects the basal ganglia to the motor cortex leads to the clinical manifestations of tremor, rigidity, bradykinesia and postural instability. Since limbic and associative loops are also affected by dopaminergic loss, cognitive and behavioural abnormalities are frequently encountered in PD patients [1-4].

Deep brain stimulation (DBS) of the subthalamic nucleus (STN) is an effective treatment to improve motor function in patients with advanced Parkinson's disease. Studies with respect to short- and long-term efficacy of STN-DBS have shown marked improvements in motor function as well as a reduction of antiparkinsonian drug treatment [5-7].

However, the effects of STN DBS on psychosocial functions are not well understood. Some studies reported positive changes in mood, depression and anxiety after surgery [8-10].

Several investigations showed adverse effects of STN DBS on mood. Although many of them were single case reports, documented mood changes include *depression *[11-13]*(hypo)mania *[14-17],*visual hallucinations *[18] and behavioural changes, like *apathy, irritability, emotional lability, hypersexuality *and *aggressiveness *[5,12,17,19]. Some behavioural abnormalities were related to electrode displacement [15,20,21] or to stimulation parameters [18,22-24]. Changes may also be related to activity modification within the basal ganglia-thalamo-cortical circuits by chronic stimulation of the STN.

Since there are hardly any long-term studies, the effects of STN DBS on mood are incompletely understood. The aim of the current study was to investigate both short- and long-term effects of STN-DBS on mood and psychosocial functions in a consecutive series of 33 patients with idiopathic PD. We analyzed patient data of the short-term follow-up that have been gathered in the study of Kalteis et al (2006) and additionally collected long-term data of the same patients [25].

## Methods

### Patients

Patients were selected for STN DBS according to the CAPSIT-PD protocol [26]. Subjects had to meet following inclusion criteria: diagnosis of idiopathic PD determined by the presence of at least two of the four cardinal motor symptoms; intractable motor fluctuations, disabling dyskinesias or freezing episodes; clear responsiveness to dopaminergic substitution therapy, demonstrated by an apomorphine test before surgery [27]; inconspicuous brain magnetic resonance imaging (MRI) scan. Exclusion criteria were previous neurosurgical history, native language other than German, substance abuse, a mini-mental state examination score (MMSE) below 24, presence of a severe psychiatric disease based on the Diagnostic and Statistical Manual of Mental Disorders (DSM-IV) and withheld informed consent.

35 consecutive patients obtained bilateral STN DBS during the assessment period. Two subjects generally refused to participate in the study. Hence, in the present investigation data of 33 PD patients (22 men, 11 women) were evaluated at baseline (see Table 1). One year after surgery patient number declined to 31 due to two deaths that were unrelated to STN DBS. During the long-term follow-up two patients withdrew informed consent, one patient died and one patient was excluded due to severe cognitive deterioration. In summary 27 subjects completed the entire investigation.

**Table 1:** Patients characteristics at baseline: mean ± SD

**Patients (n = 33)**	**m (SD)**	**range**
Age (years)	60,15 (7,88)	38 – 72
Sex (men/women)	22/11	-
Education (years)	11,09 (2,61)	8 – 17
Profession (employed/pension/household)	3/27/3	-
Disease duration (years)	13,52 (4,82)	7 – 25
Hoehn and Yahr stage	3,76 (0,56)	3 – 5
Mini Mental State Examination (MMSE)	27,85 (1,37)	25 – 30

The present study was approved by the Ethics-Committee of the Medical University of Vienna (trial registration number: 353/96).

### Surgical procedure

35 patients were treated with bilateral implantation surgery in a single operative session (Lead 3389, Medtronic Inc.). Due to a lack of cooperation one patient received the brain electrodes in two procedures. Implantation of pulse generators was performed in a second procedure one week later. We used the same surgical procedure which was described elsewhere (25).

### Assessments

To investigate short- and long-term effects of STN DBS all patients administered clinical and self-rating questionnaires prior to surgery and four times postoperatively (three, six, twelve and 36 months). To establish a baseline score, patients were assessed three times prior to electrode implantation, in fact eight to six weeks, four weeks and two weeks.

Before surgery motor symptoms were evaluated on and off medication, however psychological assessments were exclusively conducted in the on state. After surgery patients were assessed while receiving medication with stimulators turned on.

### Psychological assessment

Prior to surgery all patients underwent a comprehensive psychological evaluation. The psychological assessment included well-established self-rating scales commonly used in the assessment of mood, psychological symptoms and distress. All scales used were valid and reliable as well as recommended to evaluate treatment effects.

#### Profile of Mood Scale (POMS)

This modified version consists of 35 adjectives clustered in four subscales (Depression, Fatigue, Vigor, Irritability) by which subjects describe their mood during the week before [28].

#### Visual Analogue Scale for Well-being (VAS)

The patient has to state his present condition on a line with two extreme poles of well-being and discomfort [29].

#### Beck Depression Inventory (BDI)

The BDI is a commonly used 21-item questionnaire measuring depressive symptoms [30].

#### State-Trait Anxiety Inventory (STAI-X1/STAI-X2)

The STAI consists of two rating scales: STAI-X1 evaluates anxiety related to certain situations whereas STAI-X2 measures anxiety as a trait [31].

#### Self-Report Symptom Inventory 90 Items-Revised (SCL-90-R)

The SCL-90-R measures impairments due to somatic and psychological symptoms and distress. It comprises nine subscales and three global judgements [32].

#### Sickness Impact Profile (SIP)

The SIP is a 136-item generic quality of life instrument and consists of 12 categories that can be divided into a physical and a psychosocial domain of quality of life [33].

#### Unified Parkinson's Disease Rating Scale (UPDRS)

The severity of motor symptoms is assessed with the UPRDS-Scale [34]. Part II (activities of daily living) and III (motor examination) were evaluated for the study.

### Data analysis

Paired t-tests were performed and analyses of variance using assessment time as repeated measure to compare the time points. Due to multiple time points we chose the significance level at p ≤ 0,01.

To identify patient groups a cluster analysis was performed on the preoperative data. The squared Euclidean distance was used as a proximity measure. The number of clusters was determined by ending the hierarchical clustering with Ward's minimum variance method at a sharp increase in the error sum of squares. This criterion indicates that much of the accuracy of classification would be lost by further reducing the number of groups. Discriminant function analysis was performed to reassess the results of the cluster analysis. Additionally we conducted discriminant function analyses to predict group membership one year and three years postoperatively.

Furthermore, we calculated the Kruskal-Wallis test to compare age, education, disease duration and MMSE-score of the four subtypes. Age differences were calculated using the Chi-Quadrate test (Pearson). Results are presented as mean (± SD), otherwise indicated.

We analyzed patient data that have been collected in the study of Kalteis et al (2006) and additionally collected data three years postoperatively using the same test battery [25].

## Results

### Analysis of the preoperative Data

Baseline data were collected at three times prior to surgery (eight to six weeks, four weeks and two weeks). Since there were hardly any differences between the preoperative assessments we combined the preoperative scores to one baseline mean score. The only exception found was *Anxiety*. The subscale *Anxiety *(SCL-90-R) increased significantly from six to two weeks prior to surgery (means: 0,79 ± 0,5, 0,92 ± 0,42, T = -2,934, p = 0,007), close to the surgery date anxiety was significantly higher. This finding is not surprising as the surgical procedure was performed in awake patients off medication.

### Group classification – cluster analysis

#### Cluster analysis at baseline

Data of one patient were missed for the POMS and SIP scales; therefore we performed the analysis with 32 patients. Cluster analysis of all self rating scales at baseline identified four patient subtypes. Subtypes 1 and 2 had low well-being and higher distress level, subtypes 3 and 4 had better well-being and less distress compared to the other groups. Mean scores are listed in Table 2.

**Table 2:** Mean scores of the four patient subtypes in the self-rating scales and tests of equality of group means

**Self Rating Scales**	**cluster 1 n = 8**	**cluster 2 n = 7**	**cluster 3 n = 10**	**cluster 4 n = 7**	**Wilks' Lambda**	**F**	**p**
Depression (POMS)	18,29	12,57	9,52	3,62	,534	8,150	,000
Fatigue (POMS)	13,88	10,10	8,47	6,71	,543	7,862	,001
Vigor (POMS)	7,75	4,86	6,57	11,05	,695	4,101	,016
Irritability (POMS)	9,54	5,71	3,42	3,95	,654	4,935	,007
Well-Being (VAS)	56,64	56,70	56,90	43,85	,886	1,202	,327
Depressive Symptoms (BDI)	20,00	15,95	12,17	7,05	,442	11,771	,000
State Anxiety (STAI X1)	51,67	51,24	48,27	39,10	,592	6,436	,002
Trait Anxiety (STAI X2)	51,81	47,21	43,10	34,93	,430	12,356	,000
Somatization(SCL-90-R)	1,46	1,07	1,06	,70	,685	4,296	,013
Obsessive Compulsive (SCL-90-R)	1,09	,76	,69	,45	,644	5,163	,006
Interpersonal Sensitivity (SCL-90-R)	1,23	,73	,68	,44	,625	5,598	,004
Depression(SCL-90-R)	1,17	,80	,73	,39	,543	7,870	,001
Änxiety (SCL-90-R)	1,41	,86	,74	,43	,349	17,374	,000
Hostility (SCL-90-R)	,93	,25	,23	,36	,333	18,669	,000
Phobic Anxiety (SCL-90-R)	,82	,41	,55	,17	,725	3,532	,027
Paranoid Ideation(SCL-90-R)	1,18	,37	,60	,27	,334	18,600	,000
Psychoticism (SCL-90-R)	,67	,41	,38	,15	,500	9,331	,000
Sleep and Rest (SIP)	2,50	4,14	1,80	1,14	,532	8,211	,000
Emotional Behaviour (SIP)	1,94	2,29	1,55	,57	,703	3,935	,018
Body Care and Movement (SIP)	8,25	12,14	5,90	4,93	,628	5,531	,004
Home Management (SIP)	3,75	5,64	3,25	2,71	,729	3,466	,029
Mobility (SIP)	1,88	4,29	1,45	1,00	,590	6,478	,002
Social Interaction (SIP)	7,81	6,14	4,10	2,50	,510	8,974	,000
Ambulation (SIP)	4,38	5,43	2,90	1,79	,570	7,041	,001
Alertness Behaviour (SIP)	2,94	3,43	2,25	,86	,709	3,822	,021
Communication (SIP)	2,13	4,79	2,65	,93	,410	13,450	,000
Work (SIP)	,75	,43	,95	,57	,878	1,301	,294
Recreation and Pastimes (SIP)	4,88	4,14	3,15	2,71	,609	5,985	,003
Eating (SIP)	,44	1,79	,55	,00	,416	13,078	,000

##### Cluster 1 Very low well-being and moderate sickness impact

One fourth of the patients reported very low well-being (n = 8). They suffered from clinical relevant depressive symptoms, increased state and trait anxiety, high distress, but moderate sickness impact.

##### Cluster 2: Low well-being and a high sickness impact

22 percent described low well-being, moderate depressive symptoms and increased state and trait anxiety (n = 7). Their distress level was moderate and their sickness impact on quality of life was high.

##### Cluster 3: Slight decreased well-being and low sickness impact

31 percent of patients specified their well-being and depressive symptoms as slightly decreased (n = 10). State anxiety was increased; distress moderate and sickness impact low.

##### Cluster 4: Good well-being, little sickness impact

22 percent of patients estimated their well-being as good (n = 7). Compared to other subtypes few depressive symptoms, low anxiety, distress and sickness impact featured this cluster.

The four subtypes did not differ in age, education and disease duration (Table 3). Regarding the cognitive screening there was a trend toward significance (p = 0,018). Subtype 2 indicated the lowest MMSE score. To confirm the results of the cluster analysis we additionally performed a discriminant function analysis of the baseline data. The four cluster types again differed significantly from each other (Wilks' Lambda = 0,000, Chi-square 161,34, p = 0,000, Table 2). The procedure generated a set of discriminant functions based on linear combinations of the predictor variables that provided the best discrimination between the groups. Fisher's linear discriminant functions were generated from our sample at baseline for which group membership was defined by the cluster analysis (Table 4). Profile classification was accurately (100%) repeated in all patients which confirmed the results of the cluster analysis to the highest extent. Based on the high accordance of both statistical procedures the discriminance functions can be applied to new cases to predict group membership.

**Table 3:** Mean scores ± SD of the four patient subtypes and total (age, education, disease duration, MMSE, and distribution between the sexes)

	**cluster 1 n = 8**	**cluster 2 n = 7**	**cluster 3 n = 10**	**cluster 4 n = 7**	**total n = 32**	**p**
age	60,50 ± 6,30	64,86 ± 3,13	59,00 ± 8,33	56,43 ± 11,24	60,09 ± 8,00	0,345
education (years)	10,13 ± 1,81	11,00 ± 3,00	11,90 ± 2,85	11,14 ± 3,02	11,09 ± 2,66	0,810
disease duration (years)	10,75 ± 3,20	14,43 ± 2,64	15,50 ± 5,80	13,71 ± 5,65	13,69 ± 4,80	0,152
MMSE	28,25 ± 1,03	26,43 ± 0,98	28,50 ± 1,51	27,86 ± 1,07	27,84 ± 1,39	0,018
sex (male/female)	5/3	3/4	8/2	5/2	21/11	0,446

**Table 4:** Classification function coefficients (baseline data)

**Self rating scales (baseline)**	**Cluster types**			
	**Cluster 1**	**Cluster 2**	**Cluster 3**	**Cluster 4**
Depression/Anxiety (POMS)	-45,095	1,941	-32,259	-3,105
Fatigue (POMS)	-24,082	-40,019	-24,377	-25,016
Vigor (POMS)	-51,583	18,876	-31,280	18,696
Irritability (POMS)	75,835	-16,965	48,828	-11,831
Well-Being (VAS)	5,416	4,916	4,372	3,920
Depressive Symptoms (BDI)	3,203	-2,120	3,199	,444
State anxiety (STAI X1)	38,949	6,157	26,704	4,091
Trait anxiety (STAI X2)	-8,686	27,570	,544	21,373
Somatization (SCL-90-R)	211,872	52,274	172,128	51,987
Obsessive-Compulsive (SCL-90-R)	-1532,190	179,417	-979,230	115,394
Interpersonal Sensitivity (SCL-90-R)	41,463	512,705	133,831	382,201
Depression (SCL-90-R)	706,715	311,415	504,843	219,817
Anxiety (SCL-90-R)	-391,305	-455,959	-358,736	-359,636
Hostility (SCL-90-R)	485,733	736,884	416,970	486,936
Phobic anxiety (SCL-90-R)	249,424	-218,629	144,239	-138,369
Paranoid Ideation (SCL-90-R)	794,922	-159,428	462,165	-115,188
Psychoticism (SCL-90-R)	-521,850	-533,566	-391,216	-344,598
Sleep and Rest (SIP)	-105,772	-55,158	-84,909	-39,302
Emotional behaviour (SIP)	272,187	-57,987	166,883	-46,413
Body care (SIP)	114,992	10,448	75,009	8,841
Home management (SIP)	-23,264	-9,388	-12,351	-2,577
mobility (SIP)	-85,940	19,528	-54,831	7,265
Social interaction (SIP)	-94,910	-52,224	-76,480	-36,221
ambulation (SIP)	-164,687	123,582	-82,296	85,930
Intellectual capacity (SIP)	135,505	-32,992	87,722	-26,262
communication (SIP)	-13,535	130,803	12,335	79,070
Work (SIP)	37,462	-71,851	29,421	-63,390
Recreation and pastimes (SIP)	321,680	31,820	221,404	29,793
(Constant)	-1389,236	-1241,196	-942,598	-725,862

#### Discriminant function analysis one year after surgery

On the basis of the one-year follow-up assessment discriminance analysis again revealed significant results. Twelve months postoperatively the four profiles still significantly diverged from each other (Wilks' lambda = 0,000, Chi-square = 131,3, p = 0,000). The predicted classification remained completely unchanged in 30 patients (two patients were lost due to death). Mean scores differed significantly from baseline, in fact patients showed improvements in almost all self-rating scales one year after surgery. Despite of this clear amelioration the four patient subgroups still differed significantly. Group 1 (n = 7) and 2 (n = 6) exhibited an impaired psychosocial profile at baseline compared to the other groups (cluster 3: n = 10, cluster 4: n = 7). Group 1 and 2 improved their mean scores but both groups still demonstrated an impaired profile compared to the others.

#### Discriminant function analysis *three years *after surgery

The discriminance analysis of the three-year follow-up showed the same significant pattern as before. Even three years after surgery the four profiles remained stable (Wilks' lambda = 0,000, Chi-square = 114,23, p = 0,000). Noteworthy, mean values of the clinical self-rating scales returned back to baseline level but the profile classification still distinguished between patient groups. A total of 26 patients (one excluded because of missing baseline data) was correctly classified to cluster 1 (n = 7), cluster 2 (n = 5), cluster 3 (n = 7) and cluster 4 (n = 7).

### Pre- and postoperative changes of mood and psychosocial functions

Changes in mood and psychosocial functioning in the course of time are highlighted in detail as follows. All means (± SD), test statistics and p-values are represented in Table 5.

**Table 5:** Mean values ± standard deviation at baseline, 3, 6, 12, 36 months postoperatively

**Self Rating Scales**	**Baseline (n = 33)**	**3 months (n = 33)**	**6 months (n = 32)**	**12 months (n = 31)**	**36 months (n = 27)**	**F**	**p**
Depression (POMS)	11,09 ± 7,65	7,33 ± 7,65	7,97 ± 8,07	8,61 ± 9,22	11,85 ± 7,13	3,63	0,014
Fatigue (POMS)	9,79 ± 3,92	7,55 ± 4,99	7,84 ± 5,16	7,29 ± 5,18	9,70 ± 4,32	3,44	0,013
Vigor (POMS)	7,47 ± 3,93	8,48 ± 5,34	9,66 ± 4,92	9,29 ± 5,43	6,67 ± 4,52	3,41	0,016
Irritability (POMS)	5,57 ± 4,22	4,33 ± 4,90	4,84 ± 5,60	4,52 ± 4,75	5,33 ± 4,22	1,01	0,398
Well-Being (VAS)	53,85 ± 15,81	38,56 ± 23,96	35,44 ± 16,93	40,77 ± 20,83	48,00 ± 22,91	4,62	**0,007**
Depressive Symptoms (BDI)	13,84 ± 6,20	7,45 ± 6,36	8,47 ± 6,62	7,94 ± 5,56	13,74 ± 5,93	14,70	**<0,001**
State Anxiety (STAI X1)	47,40 ± 7,78	39,12 ± 9,50	40,44 ± 10,32	40,10 ± 9,57	45,74 ± 9,93	9,18	**<0,001**
Trait Anxiety (STAI X2)	44,26 ± 7,94	39,12 ± 10,68	38,44 ± 9,66	39,39 ± 9,40	46,26 ± 8,86	9,28	**<0,001**
Somatization(SCL-90-R)	1,06 ± 0,49	0,63 ± 0,38	0,62 ± 0,36	0,62 ± 0,43	0,82 ± 0,45	14,04	**<0,001**
Obsessive Compulsive (SCL-90-R)	0,75 ± 0,38	0,51 ± 0,39	0,56 ± 0,46	0,52 ± 0,43	0,67 ± 0,44	4,08	**0,008**
Interpersonal Sensitivity (SCL-90-R)	0,77 ± 0,46	0,50 ± 0,42	0,52 ± 0,45	0,39 ± 0,40	0,67 ± 0,47	11,32	**<0,001**
Depression(SCL-90-R)	0,79 ± 0,40	0,56 ± 0,46	0,52 ± 0,49	0,53 ± 0,48	0,87 ± 0,52	7,66	**<0,001**
Änxiety (SCL-90-R)	0,87 ± 0,43	0,32 ± 0,37	0,40 ± 0,43	0,39 ± 0,47	0,50 ± 0,48	14,40	**<0,001**
Hostility (SCL-90-R)	0,43 ± 0,35	0,28 ± 0,25	0,28 ± 0,38	0,25 ± 0,28	0,33 ± 0,38	3,14	0,032
Phobic Anxiety (SCL-90-R)	0,50 ± 0,43	0,36 ± 0,38	0,32 ± 0,41	0,36 ± 0,56	0,57 ± 0,65	2,93	0,050
Paranoid Ideation(SCL-90-R)	0,62 ± 0,42	0,45 ± 0,43	0,38 ± 0,41	0,37 ± 0,43	0,44 ± 0,49	2,51	0,052
Psychoticism (SCL-90-R)	0,41 ± 0,25	0,23 ± 0,27	0,22 ± 0,27	0,25 ± 0,37	0,36 ± 0,33	5,40	**0,001**
GSI (SCL-90-R)	0,72 ± 0,32	0,44 ± 0,30	0,47 ± 0,34	0,44 ± 0,37	0,61 ± 0,36	10,14	**<0,001**
PSDI (SCL-90-R)	1,51 ± 0,32	1,34 ± 0,30	1,31 ± 0,26	1,28 ± 0,36	1,43 ± 0,38	3,14	0,027
Physical Domain (SIP)	13,30 ± 7,59	6,06 ± 6,90	5,59 ± 6,89	6,16 ± 6,75	11,50 ± 10,42	14,46	**<0,001**
Psychosocial Domain (SIP)	11,70 ± 5,59	7,21 ± 7,17	6,19 ± 6,25	7,29 ± 8,32	13,36 ± 9,34	17,97	**<0,001**

#### POMS

The scores of three subscales (*Depression, Fatigue, Vigor*) of this self-rating scale transiently declined in the time course. However, there was just a trend towards significance (p = 0,014, p = 0,013, p = 0,016). The fourth subscale *Irritability *remained unchanged between time points.

#### Well-Being (VAS)

The estimation of well-being differed significantly (p = 0,007) in the course of time. The scores of well-being increased after the surgery up to six months, then slightly decreased but still differed from baseline. At 36 months well-being declined again and returned to baseline level.

#### BDI

Depressive symptoms diminished significantly after surgery and remained stable up to one year (p < 0,001). In the three-year follow-up the extent of depressive symptoms increased and returned to baseline level.

#### STAI

State and trait anxiety displayed significant changes (both: p < 0,001). The degree of anxiety decreased after surgery and lasted up to one year. At three years after surgery it returned to baseline level.

#### SCL-90-R

The extent of somatic symptoms (*Somatization*) declined significantly and remained stable up to one year (p < 0,001). Afterwards it increased but still significantly differed from baseline.

*Obsessive-compulsive symptoms *declined after surgery (p = 0,008). This amelioration persisted up to one year. Three years after surgery the symptoms increased and returned to baseline level.

*Interpersonal Sensitivity *improved after surgery up to one year. In the three-year follow-up it did not differ significantly from baseline (p < 0,001).

This development also applied to the subscale *Depression *(p < 0,001).

*Anxiety *(p < 0,001) significantly improved three months after surgery. One year postoperatively the score slightly augmented and further increased in the three-year follow-up but still was significantly lower compared to baseline level (p = 0,002). Prior to surgery Anxiety was the only variable that exhibited a marked increase due to the impending surgery.

*Psychotic symptoms *declined postoperatively up to 6 months. One year after surgery they slightly worsened but still differed from baseline (p = 0,001). Three years postoperatively psychotic symptoms returned to baseline level again.

The subscales *Hostility, Phobic Anxiety, Paranoid Ideation *and *Positive Symptom Distress Index *exhibited no significant changes between the assessments. The *Global Severity Index *significantly changed in the course of time (p < 0,001). After surgery the index score declined and remained stable up to one year, thereafter it increased and returned to baseline level.

#### SIP

Significant changes were also found in the psychosocial (p < 0,001) and physical domain (p < 0,001) in the course of time. Both scores improved immediately after surgery at three months and persisted up to one year. Three years postoperatively the amelioration was absent as the scores declined to baseline level.

#### UPDRS

STN DBS led to a significant improvement in activities of daily living (part II). Prior to surgery patients were assessed during the off as well as during the on state. Compared to the off state (baseline med off: 21,5 ± 6,9) there was a significant and outlasting improvement in the on state with stimulators turned on (one year: med on/stim on = 7,3 ± 3,8; three years: med on/stim on = 9,1 ± 5,7, both p = < 0,000). However, there was no significant difference between the preoperative on state (med on: 12,0 ± 5,9, p = 0,014) and postoperative scores.

STN DBS induced a clear long-term motor improvement (part III) compared to both the off and on state at baseline (baseline med off = 45,2 ± 14,0; baseline med on = 18,0 ± 9,3; 12 months: med on/stim on = 7,2 ± 7,7; 36 months: 12,0 ± 8,3, both p = < 0,001).

## Discussion

The present results suggest that prior to surgery patients with Parkinson's disease can be assigned to four distinct psychosocial profiles. Even three years after STN DBS this patient classification remained unchanged. Up to one year postoperatively there was transient improvement in mood which had no influence on group membership. The improvement disappeared in the three-year follow-up as scores returned back to baseline level. In conclusion, STN DBS only transiently improved mood and psychosocial functioning at one year. In the three-year follow-up this positive effect disappeared and returned to baseline. Thus, in the long run there was no deterioration in mood and psychosocial functioning compared to the preoperative state.

A cluster analysis revealed different psychosocial profiles that varied in well-being, depressive and anxious symptoms, distress and sickness impact. Two clusters were characterized by low well-being and moderate to high sickness impact. By comparison, the other groups were less impaired in psychosocial functioning.

Three, six and twelve months after surgery all patients improved their psychosocial characteristics but patients of cluster one and two still showed disadvantageous profiles. Three years postoperatively these ameliorations were absent but the profiles of all clusters remained relatively unchanged compared to baseline level.

Based on the patient classification of the cluster analysis it may possible to individually adjust psychosocial support to patients' needs. We assume that patients appending to cluster one or two might need more psychological support, patients of cluster three and four need less. The required amount of psychological support could be determined even prior to surgery as the psychosocial profiles remained unchanged in the course of time.

Up to one year after surgery comparisons of group means before and after surgery showed marked improvements in the majority of psychosocial variables. Well-being significantly increased, depressive and anxious symptoms, sickness impact as well as the distress level declined. These results are consistent with the findings of other groups [8,9]. Scores of three subscales (depression, fatigue and vigor) of the POMS were diminished at three, six and twelve months but these results only showed a trend towards significance. At the three-year follow-up they returned to baseline level. The bottom line is no impairment in mood and psychosocial functioning evolved in the long-term follow-up. Concordantly, no significant changes in depressive or anxiety symptoms have been reported in other studies [35,36]. Previous studies even reported worsening of depressive symptoms in a subgroup of patients [11,13]. In a recent review the overall finding was that in larger subgroups STN DBS rather had an antidepressant than depressant effect [37].

So far there are only few long-term studies with respect to mood alterations after STN DBS. One study revealed a reduction of depressive symptoms but increased apathy and thought disorders in the three-year follow-up [8]. In agreement with our results other authors found less (although not significantly) depressive symptoms one year after surgery [5]. Three and even five years after electrode implantation there was no difference to baseline level.

In line with previous studies the improvement of motor function declined to small extent but remained stable during the entire observation period [5,6,38].

In a previous study depressive symptoms were significantly improved three months after surgery, but the extent of depressive symptoms increased at twelve months and did not differ from baseline level [7]. Compared to baseline another group found no changes after STN DBS in depressive symptoms (six, 24 months and five years postoperatively) [38]. Accordingly in our study beneficial effects on well-being, depressive symptoms, state and trait anxiety, as well as on sickness impact found up to one year after STN DBS disappeared three years postoperatively.

These results suggest that motor outcome is not related to psychosocial functioning. One might ask why mood and well-being of patients declined to the initial point even though motor symptoms remained improved three years after surgery. One possible explanation of that effect stems from economic psychology. According to the prospect theory values and preferences are not entirely stable [39]. A recently gained profit or advantage soon becomes a matter of course. Based on that new status quo, losses are experienced more powerful even though they are smaller than the antecedent gains. This might be equally valid for the current results. Although ADL and motor function slightly declined in the long-term follow-up there still was a significant improvement compared to baseline. The bottom line was a net profit which may not have been perceived by patients as they only recognized the proportionally small loss. However, in conclusion STN DBS had beneficial effects on motor function and activities of daily living up to three years after surgery.

In summary, STN DBS did not induce deterioration of mood and psychosocial functions in our patients. The results showed beneficial short-term effects on mood and psychosocial functions after one year. In the three-year follow-up these beneficial effects were absent and scores returned to the preoperative level. Only amelioration of somatic symptoms and anxiety measured by the Symptom-Checklist-90-R persisted up to three years which may be related to the overall decline of motor symptoms. In this regard it is of note that a previous study reported of a gain of quality of life in parkinsonian patients twelve months after electrode implantation which however was restricted to physical aspects [40]. Mental and social quality of life did not change in the course of time. Although they used PD specific scales their findings are in line with the current results.

Based on our data we found that patients can be assigned to distinct psychosocial profiles prior to surgery. Since patient classification did not change in the course of time the individual amount of psychosocial support can eventually be anticipated before surgery in order to prevent or early detect behavioural abnormalities after STN DBS. In this regard, further studies should determine how personality traits influence subjective well-being.

Before surgery patients of cluster 1 displayed clinical relevant depression and patients of cluster 2 and 3 were moderately depressed. Their depressive pathology ameliorated up to one year and long-term results revealed at least no worsening.

Inclusion criteria of the CAPSIT-PD panel recommend surgery only for patients free of behavioural abnormalities because mainly in single case studies deterioration has been observed after surgery [26]. Besides, behavioural abnormalities mostly appeared to be transient and results are too heterogeneous to define universally valid selection criteria. Following the current inclusion criteria patients with diagnosed depression are usually meant to be excluded from STN DBS.

Despite some methodological limitations, especially the small number of patients, our findings show that STN DBS is an effective treatment PD patients with mild psychosocial disturbances. Based on the results of the cluster analysis it is possible to classify patients according to their psychosocial profile even before surgery. This allows individual determination of the extent of the collateral psychological support that is required before surgery and even in the long run.

## Conclusion

Compared to baseline no worsening in mood and psychosocial functions was found three years after STN DBS. Moreover, our results suggest that in PD patients with no to mild psychosocial and psychiatric disturbances, outcome is not affected by preoperative symptom severity. Patients with mild psychiatric problems need to receive adequate psychosocial support before and after surgery instead of being in advance excluded from STN DBS. Those patients need more attention and psychological and medical guidance in order to benefit from the motor improvement. Patients' caregivers and families need to be aware of the beneficial as well as of the possible negative effects of STN DBS. In conclusion, our study provides valuable suggestions for individual preoperative patient management and postoperative monitoring.

## Abbreviations

BDI: Beck Depression Inventory; DBS: deep brain stimulation; MMSE: Mini Mental State Exemination; PD: Parkinson's disease; POMS: Profile of Mood States; SCL-90-R: Symptom-Checklist 90-R; SIP: Sickness Impact Profile; STAI: State-Trait Anxiety Inventory; STN: subthalamic nucleus; UPDRS: Unified Parkinson's Disease Rating Scale; VAS: Visual Analogue Scale.

## Competing interests

The authors declare that they have no competing interests.

## Authors' contributions

IK: interpretation and writing, IK-E: reviewing and writing, TB: support in patient enrollment and in reviewing, DV: support in patient enrollment and in reviewing, FA: conception and design of manuscript and final approval of the version to be published.

## Pre-publication history

The pre-publication history for this paper can be accessed here:


